# National Dental Association

**Published:** 1899-09

**Authors:** 


### NATIONAL DENTAL ASSOCIATION.[1]

THE sessions of the Second Annual Meeting of the National Dental Association were held in the ball-room of the International Hotel, Niagara Falls, August 1 to 4, 1899.

The meeting was called to order at eleven A.M., Tuesday, August 1, by the President, Dr. H. J. Burkhart, of Batavia, N. Y.

After prayer by Rev. A. S. Bacon, of Niagara Falls, the President read his annual address, the Vice-President from the South, Dr. B. Holly Smith, occupying the chair. The President besought the interest of the Association in behalf of the International Dental Congress to be held in Paris in 1900; in the Army Medical Museum and Library, and in the compilation of a reliable history of the dental profession. He urged that measures be adopted to increase the membership roll of the Association. He said that the by-laws ought to be so amended that "any clean, honest, ethical dentist" may gain membership. Instead of a mere handful, there should be a membership of several thousand. The requirements

[1] Reported for the INTERNATIONAL DENTAL JOURNAL by Mrs. J. M. Walker.

at present are of such a restrictive character, and the method of electing so unsatisfactory and cumbersome, that there should be no hesitation in a radical departure from the method as presented in the constitution. The meetings of State societies are scattered throughout the whole year, and when the meeting of the National Association is at a distant interval little or no interest is felt in the election of delegates. The clause denying membership to those who without a degree have entered the profession since 1875, because there are very many who, though without a degree, are quite as well qualified as one who received his degree on the attainments required prior to 1875. Dr. Burkhart favored such revision of the constitution as would permit the election as delegates of members of any permanently organized dental societies working under the code of ethics of the National Association. He also favored the creation of an Executive Council, which shall dispose of all routine business; the sessions of the Council to be open to all members of the Association. He suggested the establishment of a scientific bureau under the auspices of the Association; recommended the correction of infirmary abuses by dental colleges; immediate measures to obtain the appointment of dentists in the army and navy, and the unification of State laws regulating the practice of dentistry. He favored a law making a license granted in one State "a passport for practice, not only on this continent, but all over the world."

On motion, the President's address was referred to a committee composed of Drs. Jas. McManus, Thos. P. Hinman, and L. P. Bethel.

Under the head of miscellaneous business, Dr. J. N. Crouse asked the privilege of the floor, and stated that news had just been received that the International Tooth Crown Company had won a suit against a New York dentist, a result which would disastrously affect every dentist in the United States unless reversal of the decision can be obtained. Dr. Crouse announced that, with the approbation of the Association, the attorney of the Dental Protective Association would be telegraphed for, and at a special meeting on Friday further explanation of the matter would be given.

The Secretary read a message of greeting from the American Dental Society of Europe, in session at Bruxelles, and introduced the delegates from that society,—Dr. Wm. Mitchell, of London, and Dr. L. C. Bryan, of Basel, Switzerland.

On motion of Dr. Patterson the courtesies of the floor were extended to these distinguished representatives of the dental profession in Europe.

The Committees on Code of Ethics and on the Journal asked further time.

The Committee on Revision of the Constitution, Dr. Thomas Fillebrown, chairman, reported the result of this work and asked an immediate vote on the portions referring to the creation and work of the Executive Council, laid over from the last annual meeting.

On motion of Dr. Hunt, the proposed amendments were ordered printed and distributed before final action is taken.

On motion of Dr. B. Holly Smith, discussion of the amended constitution was postponed until after the report of the Committee on the President's Address, as the recommendations of the President and the amendments formulated by the committees are largely in harmony, though this is a mere coincidence.

Dr. Charles McManus, chairman of the Committee on History, then read portions of a voluminous report.

On motion, the report was accepted and the committee continued, the chairman to supply the place of the lamented Dr. R. Finley Hunt, one of the most active workers on the committee, who died May 21, 1898, at the venerable age of eighty-one years.

The various officers of the Association then read their reports, that of the treasurer showing the receipt of annual dues to the amount of $1247, with a balance on hand of $881.03.

Dr. M. F. Finley, chairman, read the report of the Committee on Dentists in the Army and Navy. He said that it was better to have no legislation than that of political machinery, and that no expedient plan had yet been devised of attaining the desired object. The Surgeon-General is not opposed to the innovation, but he is not impressed with the importance of offering such inducements as would secure the most efficient men.

On motion, the report was accepted and referred to the Committee on the President's address.

On motion of Dr. Patterson, of the Executive Committee, a resolution was adopted rescinding the vote of censure of the New Jersey State Society, passed at the Omaha meeting. The resolution was signed by Drs. Patterson and Finley, of the Division on Credentials.

On motion, adjourned to 7.30 P.M.

(To be continued.)

